# Predictors for MTB Culture-Positivity among HIV-Infected Smear-Negative Presumptive Tuberculosis Patients in Uganda: Application of New Tuberculosis Diagnostic Technology

**DOI:** 10.1371/journal.pone.0133756

**Published:** 2015-07-29

**Authors:** Lydia Nakiyingi, Bareng A. S. Nonyane, Willy Ssengooba, Bruce J. Kirenga, Damalie Nakanjako, Gloria Lubega, Pauline Byakika-Kibwika, Moses L. Joloba, Jerry J. Ellner, Susan E. Dorman, Harriet Mayanja-Kizza, Yukari C. Manabe

**Affiliations:** 1 Infectious Diseases Institute, Makerere University College of Health Sciences, Kampala, Uganda; 2 Department of Medicine, School of Medicine, Makerere University College of Heath Sciences, Kampala, Uganda; 3 Department of International Health, Johns Hopkins Bloomberg School of Public Health, Baltimore, Maryland, United States of America; 4 Department of Medical Microbiology, Makerere University College of Heath Sciences, Kampala, Uganda; 5 Division of Infectious Diseases, Department of Medicine, Johns Hopkins University School of Medicine, Baltimore, Maryland, United States of America; 6 Boston Medical Center, Boston University School of Medicine, Boston, MA, United States of America; National Institute of Infectious Diseases, JAPAN

## Abstract

**Background:**

The existing World Health Organization diagnostic algorithms for smear-negative TB perform poorly in HIV-infected individuals. New TB diagnostics such as urine TB lipoarabinomannan (LAM) could improve the accuracy and reduce delays in TB diagnosis in HIV-infected smear-negative presumptive TB. We sought to determine predictors for MTB culture-positivity among these patients.

**Methods:**

This study was nested into a prospective evaluation of HIV-infected outpatients and inpatients clinically suspected to have TB who were screened by smear-microscopy on two spot sputum samples. Data on socio-demographics, clinical symptoms, antiretroviral therapy, CXR, CD4 count, mycobacterial sputum and blood cultures and TB-LAM were collected. Logistic regression and conditional inference tree analysis were used to determine the most predictive indicators for MTB culture-positivity.

**Results:**

Of the 418 smear-negative participants [female, 64%; median age (IQR) 32 (28-39) years, median CD4 106 (IQR 22 - 298) cells/mm^3^], 96/418 (23%) were sputum and/ or blood culture-positive for *Mycobacterium tuberculosis* (MTB) complex. Abnormal CXR (aOR 3.68, 95% CI 1.76- 7.71, p=0.001) and positive urine TB-LAM (aOR 6.21, 95% CI 3.14-12.27, p< 0.001) were significantly associated with MTB culture-positivity. Previous TB treatment (aOR 0.41, 95% CI 0.17-0.99, p=0.049) reduced the likelihood of a positive MTB culture. A conditional inference tree analysis showed that positive urine TB-LAM and abnormal CXR were the most predictive indicators of MTB culture-positivity. A combination of urine TB-LAM test and CXR had sensitivity and specificity of 50% and 86.1% respectively overall, and 70.8% and 84.1% respectively among those with CD4<100 cells/mm^3^.

**Conclusions:**

A positive urine TB-LAM test and an abnormal CXR significantly predict MTB culture-positivity among smear-negative HIV-infected presumptive TB patients while previous TB treatment reduces the likelihood of a positive MTB culture. Validation studies to assess the performance of diagnostic algorithms that include urine TB-LAM in the diagnosis of smear-negative TB in HIV-infected individuals are warranted.

## Introduction

Tuberculosis (TB) is a major cause of death among HIV-infected patients [1–3]. Early diagnosis of TB is essential for prompt initiation of therapy to reduce TB-associated morbidity and mortality as well as transmission. Despite the current wave of rapid molecular TB diagnostics, sputum smear microscopy continues to be the most available and used TB diagnostic in many SSA countries [4, 5]. Several studies in sub-Saharan Africa (SSA) have found an increased prevalence of sputum smear-negative TB in HIV-infected patients [6] which is associated with high morbidity and mortality [7–10] as a consequence of delayed or missed diagnosis. This has emphasized the need for more rapid, sensitive and affordable TB diagnostic tools. There are few inexpensive and easy to use alternatives to sputum smear microscopy in resource limited settings (RLS) [11–14]. *Mycobacterium tuberculosis* (MTB) culture, which is the gold standard for diagnosis, is not widely available due to technical and biosafety requirements [12, 13], while the use of molecular tests such as Xpert MTB/Rif test is limited by cost [15–17]. The WHO recommended diagnostic algorithms for smear-negative pulmonary TB (PTB) [18] are limited by the poor diagnostic performance particularly in HIV-infected individuals [11, 19–22] and the long duration required to establish a diagnosis (11 to 34 days) [23, 24], which poses risks of disease progression, transmission and loss to follow-up. Several validation studies have found poor performance of the existing algorithms for smear-negative TB diagnosis especially in HIV [19–22]. For instance, the sensitivity and specificity of the diagnostic algorithm for smear-negative PTB were 38.1% and 74.5% respectively in a Tanzania study [22] and 55% and 72.9% respectively in Kenya [21]. In addition, several reports indicate that the guidelines are not always followed by clinicians [11, 25]; with majority empirically initiating TB treatment. In Botswana [25] for instance, laboratory delays and inability by patients to produce sputum were cited as reasons for not following the guidelines. In our recent study [26], empirical TB treatment initiation resulted in over-treatment of HIV-infected patients. Strategies to improve the diagnosis of smear-negative TB in HIV-infected individuals, particularly those that would reduce laboratory delays or provide alternatives to sputum production in HIV are warranted.

The lateral flow urine determine TB lipoarabinomannan (LAM) antigen test (herein referred to as urine TB-LAM test) is a new point of care antigen test which provides results within less than an hour of sample collection and has been shown to perform best among HIV-infected patients with advanced immune-suppression [27–29]. In addition, the TB-LAM test utilizes urine which is readily available and easy to collect in HIV-infected individuals. Urine TB-LAM test has the potential to improve accuracy and to reduce delays associated with the current smear-negative TB diagnostic algorithms but has not been evaluated as a possible predictor for MTB culture-positivity among smear-negative HIV-infected presumptive TB patients.

We previously showed that in HIV-infected adults with CD4≤100, the urine TB-LAM tests detected over half of culture-positive TB patients without need for equipment or reagents. Our report considered HIV-infected adults with TB symptoms and/or signs irrespective of their sputum-smear status [27]. In this study, we sought to determine the factors that could predict MTB culture-positivity among smear-negative HIV-infected presumptive TB patients by applying the urine TB-LAM test as a new point of care technology in TB diagnosis. The aim is to develop strategies to improve the diagnosis of smear-negative TB in HIV-infected patients and to reduce related diagnostic delays through possible application of the new technology in the development of smear-negative TB diagnostic criteria in HIV-infected individuals.

## Methods

### Study design and setting

We performed secondary analysis of data collected from participants enrolled in a TB diagnostic accuracy study of the lateral flow TB-LAM test for the diagnosis of TB among HIV-infected presumptive TB patients [27]. Participants for this study were recruited from the outpatient adult Infectious Diseases Institute (IDI) Clinic [30] and the inpatient wards of the Mulago National Referral Hospital, Kampala Uganda between January and November 2011.

### Patient recruitment and study population

Patient recruitment has been described in detail previously [27]. In summary, HIV-infected patients aged ≥18 years and clinically suspected to have active TB were recruited. A patient was suspected to have active TB (presumptive TB) if he/she reported having at least one of the following symptoms: cough, fever, drenching night sweats or weight loss [3]. Patients were excluded if they had taken anti-TB medication for more than two days within 60 days prior to enrolment.

At enrolment, participants were interviewed to obtain socio-demographic and medical information before study-specific specimens were collected. They provided two spot sputum samples for direct fluorescence microscopy (FM) and Ziehl-Neelson (ZN) microscopy; mycobacterial growth indicator tube (MGIT) and Lowenstein-Jensen (LJ) sputum cultures. Sputum induction using 7% nebulized hypertonic saline was performed for participants who were unable to spontaneously expectorate sputum. Blood was collected from each participant for mycobacterial blood cultures and to determine the CD4 cell count. Urine was collected for TB-LAM testing using a lateral flow assay (TB-LAM Alere, Waltham, MA, USA). Chest X-ray was obtained for male participants and non-pregnant women. Participants were classified as sputum smear-negative if they were smear-negative on both ZN and FM microscopy and these were considered for this analysis. MTB culture-positives were MTB positive on any of the sputum or blood mycobacterial cultures.

### Laboratory procedures

Details of the laboratory procedures for the TB tests including smear microscopy, sputum and blood TB culture as well as urine TB-LAM test have been described [27]. In summary, for urine TB-LAM test, 60 μl of urine was pipetted onto the sample pad. According to the manufacturer’s instructions, the strip was read 25 minutes later by two different technicians independently who compared the test strips with the reference card provided by the manufacturer and graded the result from 1+ to 5+. A result was considered positive if the band was graded as 2+ or above. CD4 cell counts were performed at the College of American Pathologists (CAP)-certified laboratory at the IDI [30].

All study TB laboratory results except for urine LAM which was an investigational test were made available to the attending clinicians. Discharged participants were contacted by telephone to deliver results and those whose TB tests were positive were requested to return for treatment. Participants whose TB results were positive but could not be contacted by phone were visited at their homes. MTB positive patients by smear and/or culture were immediately initiated on TB treatment by the attending clinician according to Uganda Ministry of Health TB and Leprosy program guidelines [31].

### Statistical analysis

Statistical analyses were performed using STATA (Stata Corp. STATA 12.0, college station, Texas 77845 USA.) and R version 3.1.1 (the R Foundation for Statistical Computing 2014).

The primary outcome was MTB culture status (on blood and/or sputum TB cultures) of sputum smear-negative HIV-infected presumptive TB participants, classified as positive or negative. Continuous variables were summarized using medians and inter-quartile ranges (IQR) while categorical variables were summarized using frequencies, percentages and proportions. Using Wilcoxon rank sum test for continuous variables and either chi-square test or Fisher’s Exact test for categorical variables as appropriate, we compared the characteristics of the study population by the strata of the primary outcome.

To identify predictors of MTB culture positivity among sputum smear-negative HIV-infected presumptive TB patients, bivariate and multivariate logistic regression models were built and results presented as unadjusted and adjusted odds ratio (OR) with 95% confidence intervals (95% CI). The modeling process involved selecting all factors from bivariate analysis that had a p-value of ≤ 0.2 and those of known clinical significance for inclusion in the initial multivariate logistic regression model. Using a systematic backward approach, non-significant variables were removed from the model until no further variables were eligible for removal to arrive at the final model. ART treatment and CD4 cell count were retained in the model at all levels due to their clinical significance. A p-value of < 0.05 in the final model was considered statistically significant. In order to determine the relative predictive power of the variables considered in the multivariable model, we fitted a conditional inference tree model [32, 33]. The model included only the smear-negative participants with complete records. This model was fitted using the ‘party’ package in R.

### Ethical considerations

The study was approved by the Scientific Review Board of the Infectious Diseases Institute (IDI), the Institutional Review Board (IRB) of the Joint Clinical Research Centre, Kampala, Uganda and the Uganda National Council for Science and Technology. All participants provided written informed consent before participation in the study. All consent forms were approved by the above mentioned ethics committees and IRBs.

## Results

### Baseline characteristics

**Fig 1:**
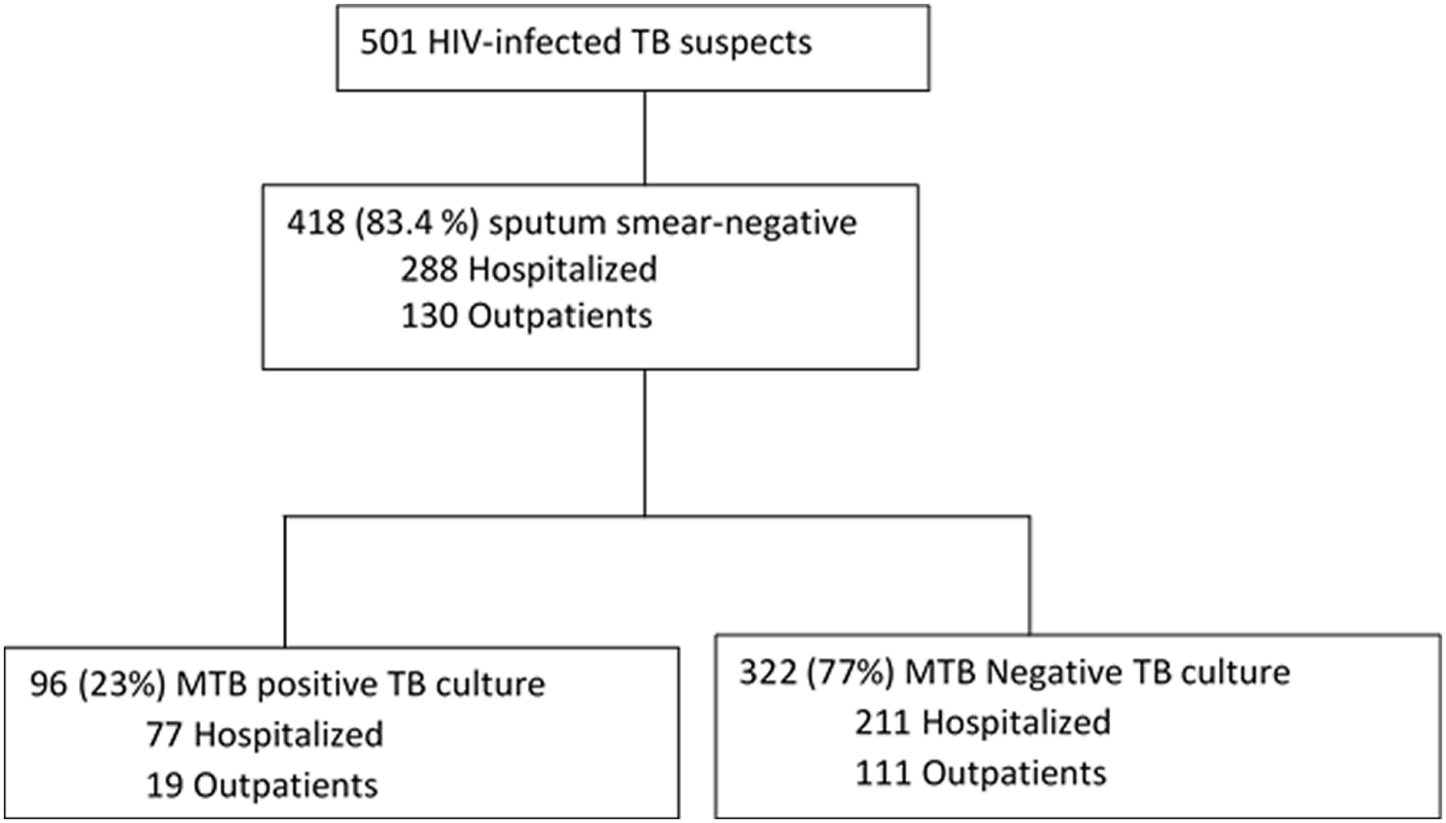
Participant flow diagram and MTB culture results distribution.

Of the 501 HIV-infected presumptive TB patients who provided sputum samples for microscopy, 418 (83.4%) were sputum smear- negative and eligible for this analysis **(** Fig 1). Of the 418 sputum smear-negative participants, 69% (288/418) were inpatients, 64% (267/418) were female; median age was 32 (IQR 28–39) years and median CD4 was 106 (IQR 22–298) cells/mm^3^.

Of the 418 sputum smear-negative participants, 96/418 (23%) were positive for MTB complex on blood and/or sputum TB cultures; of which, 55/96 (57.3% were MTB culture positive on sputum alone, 35/96 (36.5%) were positive on both sputum and blood cultures while 6/96 (6.3%) were positive on blood culture alone. The median CD4 cell count was significantly lower in the MTB culture-positive compared to the culture-negative participants [60 (IQR 14–152) versus 127 (IQR 26–329) cells/mm^3^, p = 0.001 respectively]. Majority of the MTB culture-positives were inpatients (80%). A comparison of clinical and laboratory characteristics between MTB culture-positive and negative participants is shown in Table 1.

**Table 1:** Clinical and socio-demographic characteristics of smear-negative HIV- infected presumptive TB patients.

Characteristics	Total n = 418	Culture-negative n = 322	Culture-positive n = 96	P value
Gender: Female	267 (63.9)	215 (66.8)	52 (54.2)	**0.024**
Age (years): Median (IQR)	32 (28–39)	32 (28–39)	32 (28–38)	0.116
Inpatients	288 (68.9)	211 (65.5)	77 (80.2)	**0.006**
* *CD4 count*, *cells/μl (N = 413)*				
Median CD4 (IQR)	106 (22–298)	127 (26–329)	60 (14–152)	**0.001**
<50	158 (38.3)	114 (35.7)	44 (46.8)	**0.002**
50–99	43 (10.4)	29 (9.1)	14 (14.9)	**0.003**
100–199	70 (17.0)	51 (16.0)	19 (20.2)	**0.008**
200–350	61 (14.8)	52 (16.3)	9 (9.6)	0.378
>350	81 (19.6)	73 (22.9)	8 (8.5)	reference
***Treatment history***				
Previous TB	76 (18.2)	68 (21.1)	8 (8.3)	**0.004**
Not on ART treatment	261 (62.4)	193 (59.9)	68 (70.8)	0.053
Cotrimoxazole prophylaxis	390 (93.3)	302 (93.8)	88 (91.7)	0.465
***Symptoms***				
**Cough duration in weeks (IQR)	4 [2–8)	4 [2–6)	4 [2–8)	0.204
*Drenching night sweats (N = 407)	303 (74.5)	224 (71.1)	79 (85.9)	**0.004**
*Anorexia (n = 404)	303 (75.0)	224 (71.3)	79 (87.8)	**0.001**
Fever	379 (90.7)	292 (90.7)	87 (90.6)	0.986
Weight (kg): Median (IQR)	50 (45–55)	50(44–56)	51(45–55)	0.944
***Investigations***				
*Abnormal CXR (N = 381)	43 (11.3)	22 (7.5)	21 (24.4)	**0.000**
*Positive urine TB-LAM (N = 417)	58(13.9)	22(6.9)	36(37.5)	**0.000**

* means the number of variables is less than the total N = 418. These were missing data due to unrecorded data and unperformed study tests. CXR was considered abnormal if the study clinician reported the CXR as highly suggestive of TB.

**means period for which the patient has had the current cough at the time of enrolment.

**Abbreviations**: TB, tuberculosis; ART, antiretroviral therapy; CXR, chest X-ray; LAM, lipoarabinomannan; IQR, inter-quartile range

### Predictors of MTB culture-positive, smear-negative TB

**Fig 2:**
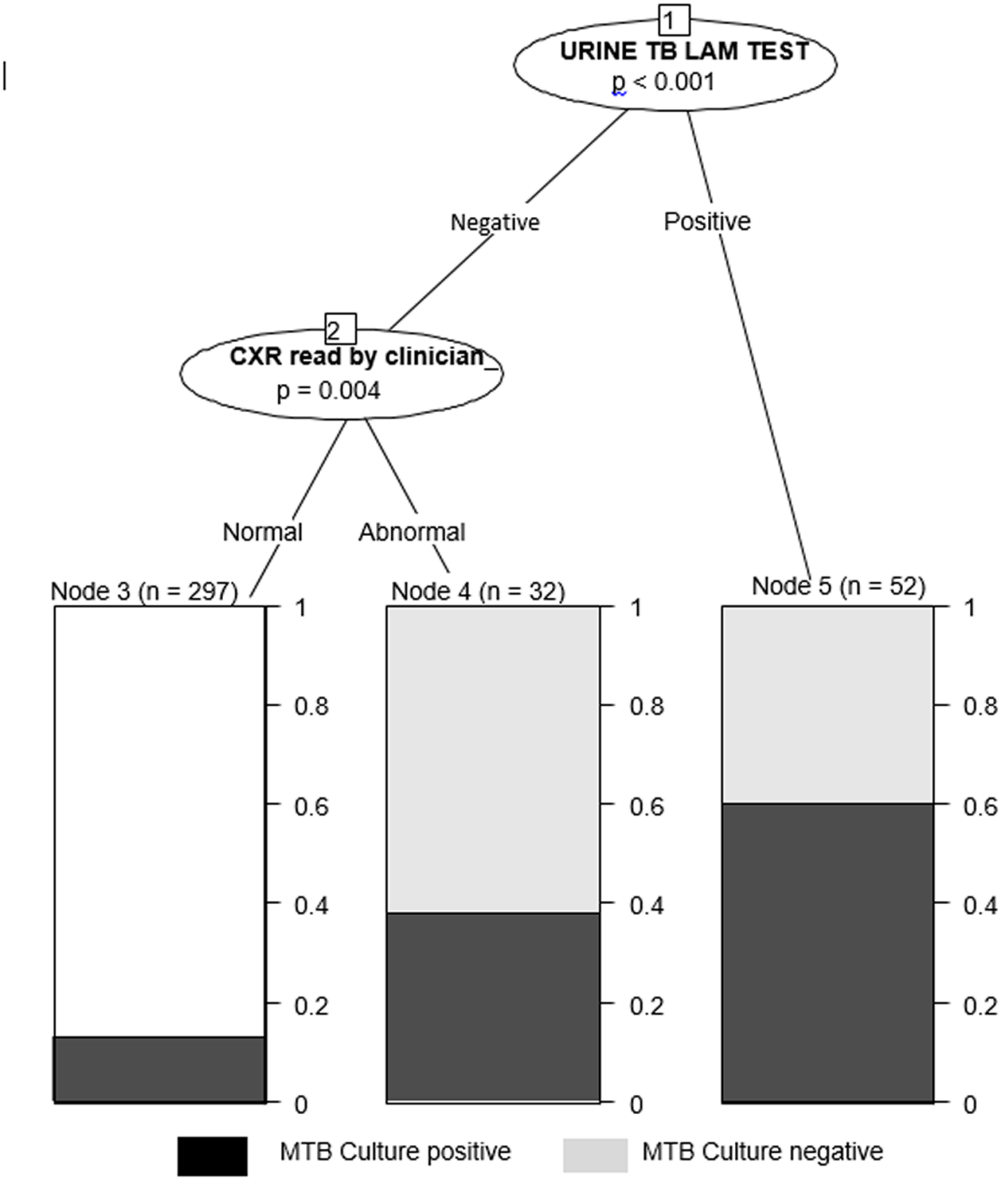
A plot of a conditional inference tree showing the most predictive indictors for MTB culture-positivity. The model was fitted on the 381 participants with complete records.

Results of bivariate analysis are presented in Table 2. On multivariate logistic regression analysis (Table 2), presence of an abnormal CXR (aOR 3.68, 95% CI 1.76–7.71, p = 0.001) and a positive urine TB-LAM test (aOR 6.21, 95% CI 3.14–12.27, p< 0.001) were significantly associated with MTB culture-positivity among smear-negative HIV-infected presumptive TB patients. On the other hand, previous TB treatment (aOR 0.41, 95% CI 0.17–0.99, p = 0.049) reduced the likelihood of a positive TB culture. The conditional inference tree model to determine the ranking of the factors showed that the urine TB-LAM was the most predictive of MTB culture positivity, followed by a CXR in this cohort of patients based on Bonferroni-adjusted p-values (p<0.001 for urine TB-LAM and p = 0.004 for CXR). A plot of the fitted conditional inference tree (Fig 2). From the model, of the 52 participants that were declared positive by the urine TB-LAM test, 31(59.6%) were true MTB culture positives. Of the 329 participants that had been declared negative by urine TB-LAM test, the CXR found an additional 12 true MTB culture positives.

**Table 2:** Logistic regression analysis for predictors of MTB culture-positive TB in sputum smear-negative HIV-infected presumptive TB**.

Characteristics	crude OR (95%CI)	P-value	Adjusted OR (95%CI)	P-value
Female gender	0.59(0.37–0.94)	0.025	-	-
Age per 5 year increase	0.91 (0.79–1.05)	0.194	-	-
Hospitalization	2.13 (1.23–3.70)	0.007	-	-
CD4(cells/μl); per 50 cells increase	0.87 (0.80–0.93)	0.000	0.93 (0.86–1.01)	0.070
Previous TB treatment	0.34 (0.16–0.73)	0.006	**0.41 (0.17–0.99)**	**0.049**
Not on ART treatment	1.62 (0.99–2.66)	0.054	1.39 (0.76–2.53)	0.284
Cotrimoxazole prophylaxis	.73 (0.31–1.71	0.467	-	-
Cough duration (weeks)	1.02 (0.99–1.06)	0.207	-	-
Weight (per kg increase)	1.00 (0.98–1.02)	0.944	-	-
Abnormal CXR	4.01 (2.08–7.73	0.000	**3.68 (1.76–7.71)**	**0.001**
Positive urine TB-LAM	8.15 (4.48–14.84	0.000	**6.21 (3.14–12.27)**	**0.000**

** N = 381 with complete records.

The model adjusted for CD4 cell count, previous TB treatment, antiretroviral therapy, CXR findings, ART therapy and urine TB-LAM test

**Abbreviations**: TB, tuberculosis; ART, antiretroviral therapy; CXR, chest X-ray; LAM, lipoarabinomannan; IQR, inter-quartile range; OR, odds ratio; CI, confidence intervals.

### Accuracy of urine TB-LAM in the diagnosis of smear-negative–culture positive TB in HIV

The accuracy values (Tables 3 and 4) for urine TB-LAM test were calculated using MTB culture performed on blood and/or sputum specimens as the reference standard. The urine TB-LAM test had a sensitivity and specificity of 37.5% and 93.1% respectively among all smear-negative participants and 55.2% and 89.5% respectively among participants with CD4<100 cells/mm^3^. A combination of urine TB-LAM test and CXR had sensitivity and specificity of 50% and 86.1% respectively overall, and 70.8% and 84.1% respectively among those with CD4<100 cells/mm^3^ (Table 3). The performance indices among the inpatient and the outpatient participants are shown in Table 4.

**Table 3:** Performance of the urine TB-LAM test in the diagnosis of smear-negative TB stratified by CD4 cell count.

	Overall accuracy (95% CI)	Accuracy among patients with CD4<100 (95% CI
**Urine TB-LAM test**	**N = 417**	**N = 201**
Sensitivity	37.5 (27.8–48.0)	55.2 (41.5–68.3)
Specificity	93.1 (89.8–95.7)	89.5 (83.3–94.0)
PPV	62.1 (48.4–74.5)	68.1 (52.9–80.9)
NPV	83.3 (79.0–87.0)	83.1 (76.2–88.7)
LR (positive)	5.47 (3.39–8.83)	5.26 (3.09–8.95)
LR (negative)	0.67 (0.57–0.79)	0.50 (0.37–0.67)
**Combination of Urine TB-LAM test and CXR**	**N = 381**	**N = 174**
Sensitivity	50.0 (39.0–61.0)	70.8 (55.9–83.0)
Specificity	86.1 (81.6–89.8)	84.1 (76.6–90.0)
PPV	51.2 (40.0–62.3)	63.0 (48.7–75.7)
NPV	85.5 (81.0–89.3)	88.3 (81.2–93.5)
LR (positive)	3.60 (2.52–5.13)	4.46 (2.87–6.94)
LR (Negative)	0.58 (0.47–0.72)	0.35 (0.22–0.54)

**Abbreviations**: TB, tuberculosis; LAM, lipoarabinomannan; CXR, Chest X-ray; PPV, positive predictive value; NPV, negative predictive value; LR, likelihood ratio

**Table 4:** Performance of the urine TB-LAM test in the diagnosis of smear-negative TB among inpatients and outpatients stratified by CD4 cell count.

	Overall accuracy(95% CI)		Accuracy among patients with CD4<100 (95% CI)	
	Inpatients	outpatients	Inpatients	Outpatients
**Urine TB-LAM**	**N = 287**	**N = 130**	**N = 169**	**N = 32**
Sensitivity	39.0 (28.0–50.8)	31.6 (12.6–56.6)	54.2 (39.2–68.6)	60.0 (26.2–87.8)
Specificity	90.0 (85.1–93.7)	99.1 (95.1–100.0)	87.6 (80.4–92.9)	100.0 (84.6–100.0)
PPV	58.8 (44.2–72.4)	85.7 (42.1–99.6)	63.4 (46.9–77.9)	100.0 (54.1–100.0)
NPV	80.1 (74.4–85.0)	89.4 (82.6–94.3)	82.8 (75.1–88.9)	84.6 (65.1–95.6)
LR (positive)	3.90 (2.38–6.38)	35.05 (4.47–275.1)	4.37 (2.55–7.50)	N/A
LR (negative)	0.68 (0.56–0.82)	0.69 (0.51–0.94)	0.52 (0.38–0.72)	0.40 (0.19–0.85)
**Combination of Urine TB-LAM and CXR**	**N = 256**	**N = 125**	**N = 144**	**N = 30**
Sensitivity	52.9 (40.4–65.2)	38.9 (17.3–64.3)	71.8 (55.1–85.0)	66.7 (29.9–92.5)
Specificity	84.0 (78.0–89.0)	89.7 (82.3–94.8)	81.0 (72.1–88.0)	100.0 (83.9–100.0)
PPV	54.5 (41.8–66.9)	38.9 (17.3–64.3)	58.3 (43.2–72.4)	100.0 (54.1–100.0)
NPV	83.2 (77.1–88.2)	89.7 (82.3–94.8)	88.5 (80.4–94.1)	87.5 (67.6–97.3)
LR (positive)	3.32 (2.23–4.94)	3.78 (1.69–8.46)	3.77 (2.43–5.86)	N/A
LR (Negative)	0.56 (0.43–0.73)	0.68 (0.47–0.99)	0.35 (0.21–0.58)	0.33 (0.13–0.84)

**Abbreviations**: TB, tuberculosis; LAM, lipoarabinomannan; CXR, Chest X-ray; PPV, positive predictive value; NPV, negative predictive value; LR, likelihood ratio

## Discussion

Diagnosis of smear-negative TB in HIV-infected individuals is difficult and is often associated with delays. New TB diagnostics techniques such as the urine TB-LAM antigen test have a potential to improve accuracy and to reduce delays in smear-negative TB diagnosis in HIV. We aimed to identify factors that could predict MTB culture-positivity among sputum smear-negative HIV-infected presumptive TB patients in a high prevalence TB/HIV setting and also assessed the accuracy of urine TB-LAM test in smear-negative TB diagnosis. In our cohort, the prevalence of culture-positive TB was high at 23% of the smear-negative HIV-infected presumptive TB patients. The predictors for MTB culture-positivity among smear-negative HIV-infected patients were; positive urine TB-LAM and an abnormal CXR read as highly suggestive of TB by a clinician. Previous TB treatment reduced the likelihood of finding a positive MTB culture. Positive urine TB-LAM test followed by CXR were the most predictive combination and would reduce the total number of CXRs that would have to be performed. CXR facilities are not readily available in many lower level health facilities in RLS and where available, they may not be affordable and maintenance is costly.

Similar to earlier reports from other sub-Saharan African studies [6, 34, 35], we found a high prevalence of MTB culture-positive smear-negative TB among HIV-infected presumptive TB patients and these MTB culture positive participants had immunologically advanced HIV disease as shown by the low median CD4 cell counts obtained. HIV-infected patients with advanced immunosuppression often have paucibacillary TB which reduces the sensitivity of sputum smear microscopy, requiring TB diagnostic tests with higher sensitivity such as TB culture. Our study found that abnormal CXR was independently associated with a positive MTB culture in smear-negative HIV-infected presumptive TB patients. This finding corroborates with several earlier studies [20, 24, 36–39] that report CXR and other clinical symptoms as important predictors for TB in sputum smear-negative presumptive TB patients. On the other hand, several other studies [21, 22, 40, 41] have reported poor diagnostic performances of clinical and radiological characteristics in smear-negative TB diagnosis. The variation in performance could be due to the differences in the study settings and/or populations. Studies performed among largely HIV-infected populations have reported poorer performances [21, 22, 40, 41] when compared with studies that included very few or no HIV-infected patients [42, 43].

A positive urine TB-LAM test was another independent predictor for MTB culture-positivity among HIV-infected smear-negative participants in our study, with higher accuracy values seen among participants with CD4<100 cells/mm^3^. Our findings are in agreement with earlier reports that urine TB-LAM performs much better among HIV-infected individuals with advanced HIV disease [27–29]. This could be explained in part by the fact that HIV-infected presumptive TB patients who have negative sputum smears could have other forms of TB including extra pulmonary and/or disseminated TB that is diagnosed by the urine TB-LAM test. However, the sensitivity of urine TB-LAM increases at the expense of the specificity in patients with CD4 <100 cell count. This could be due to other comorbidities mimicking TB that could occur among patients with advanced HIV disease or that sputum and blood mycobacterial cultures are an incomplete gold standard. A significant association was found between previous TB treatment and MTB culture positivity on logistic regression, with previous TB being associated with reduced likelihood of MTB culture positivity. This highlights the clinical issue of patients who have previously suffered from TB presenting with respiratory symptoms (probably due to post-TB complications) who are often misdiagnosed as presumptive TB.

Our findings suggest that a combination of urine TB-LAM test followed by a CXR significantly improves the accuracy of TB diagnosis in smear-negative HIV-infected patients, especially when CD4 counts are < 100cells/mm^3^. Almost two thirds of MTB culture-positive smear negative HIV-infected patients with CD4< 100cells/mm^3^ in our study were diagnosed by a combination of urine TB-LAM and CXR with a high negative predictive value. Use of this proposed algorithm would reduce on the inaccuracy associated with empirical TB treatment initiation as often used in smear-negative HIV-infected individuals [26]. Information on whether TB treatment initiation in HIV- infected patients with the proposed algorithm would improve patients’ outcome or not would be important to guide its clinical application. Therefore, our data provide rationale for future research on assessment of the impact of TB treatment initiation among HIV-infected patients with positive urine TB-LAM and abnormal CXR on patients’ outcome.

Further, the application of the urine TB-LAM based algorithm suggested from our findings would be limited to low-income settings where the highly accurate but costly molecular assays are not readily available for use in TB diagnosis. In an earlier report [44], urine TB-LAM and sputum Xpert MTB/Rif testing were complementary to each other and therefore, the algorithms may be useful in settings where use of Xpert MTB/Rif test is limited by cost and unavailability. In addition, the urine TB-LAM test does not identify multiple drug resistant (MDR) TB strains, thus its use may be limited to settings where the prevalence of MDR is low, as is the case in our study setting [45].

Our study had some limitations. First, the mycobacterial blood and sputum cultures may have failed to detect some participants with TB and some culture-negative patients could have been misclassified as not TB. This may have underestimated the association between the predicting factors and TB disease in this population as well as the accuracy of the suggested algorithm. Secondly, we did not follow-up the MTB culture-negative participants to obtain information on empirical TB treatment.

Despite these limitations, our study has important strengths. In order to accurately classify participants as having TB or not, we performed both solid and liquid MTB cultures in addition to TB blood cultures, making the findings more reliable and accurate. The classification of participants as smear-negative presumptive TB was based on both ZN and FM microscopy, also making our study population definition and findings more accurate and reliable. Further, we report findings from a population that includes both outpatients and inpatients making our findings generalizable and relevant to most HIV/TB prevalent settings.

In conclusion, smear-negative culture-positive TB is common in this setting. Positive urine TB-LAM test and abnormal CXR significantly predict MTB culture-positive TB among smear-negative HIV-infected presumptive TB patients while previous TB treatment reduces the likelihood of a positive MTB culture. Positive urine TB-LAM test followed by CXR are the two most predictive factors for MTB culture positivity. Urine TB-LAM test may be applied in the development of diagnostic algorithms for smear-negative TB in HIV. We recommend validation studies to assess the performance of diagnostic algorithms that include urine TB-LAM test in the diagnosis of smear-negative TB in HIV-infected individuals.
